# Type 2 diabetes care: Improvement by standardization at a diabetes rehabilitation clinic. An observational report

**DOI:** 10.1371/journal.pone.0226132

**Published:** 2019-12-12

**Authors:** Helmuth Haslacher, Hannelore Fallmann, Claudia Waldhäusl, Edith Hartmann, Oswald F. Wagner, Werner Waldhäusl

**Affiliations:** 1 Department for Laboratory Medicine, Medical University of Vienna, Waehringer Guertel, Vienna, Austria; 2 Rehabilitation Clinic for Diabetes and Metabolic Diseases, Moorbad Neydharting, Neydharting, Neydharting, Austria; 3 Department of Radiotherapy, Medical University of Vienna, Waehringer Guertel, Vienna, Vienna, Austria; 4 Department of Medicine III, Medical University of Vienna, Waehringer Guertel, Vienna, Vienna, Austria; West Virginia University, UNITED STATES

## Abstract

**Background:**

Outcome of type 2 diabetes care depends on the acceptance of self-responsibility by informed patients, as treatment goals will otherwise be missed.

**Aims and methods:**

This pre/post-observational report describes the clinical outcome of type 2 diabetes care in patients with type 2 diabetes (N =930) admitted consecutively to a diabetes rehabilitation clinic (DRC) between June 2013, and June 2016, where they were exposed to standardized lifestyle modification with meals low in salt and rich in vegetables and fruits, totaling 1,200 to 1,600 kcal/d, and an add-on exercise load equivalent to 400–600 kcal/d.

**Results:**

At admission, patients presented with multiple treatment modes, elevated HbA_1c_ levels (7.6±1.5%, 60±16 mmol/mol), a high prevalence of co-morbidities dominated by obesity (79%), a low rate of influenza and pneumococcal immunization (<9%) and underuse of lipid-lowering drugs (-29%). Analysis of clinical and metabolic outcome after 3 weeks shows that simple standardization of and better adherence to treatment recommendations improved (p<0.0001) glucose (HbA_1c_ -0.4±0.4%) and lipid metabolism (LDL/HDL ratio, -0.58±0.03), permitting a 39% reduction in insulin dosage, omission of insulin in 36/232 patients and omission of oral antidiabetic drugs (OADs) other than metformin and DPP4-inhibitors, while the use of GLP-1 analogs doubled to 5.2%. Improved outcome was independent of treatment strategy and more marked at initially high HbA_1c_ at costs less than 25% of those encountered at a standard hospital.

**Conclusions:**

Our observations support the clinical notion that adherence to basic treatment recommendations is indispensable in type 2 diabetes care if metabolic and clinical treatment goals are to be met, and if inappropriate add-on over-medicalization with OADs and/or insulin is to be avoided. To this end, *‘imprinting’* patients at a DRC could be of considerable help.

## Introduction

Type 2 diabetes is a major health burden. Its prevalence increases worldwide [1] based on a polygenic background [2] due to environmental and behavioral risk factors [3] causing energy imbalance, diabesity, and insulin insensitivity [4].

Treatment of type 2 diabetes requires a multifactorial integrated treatment regimen [5] including permanent self-control of energy balance, i.e., of daily food intake and exercise load. To meet metabolic goals set [6], and to avoid the development of microvascular [7] as well as macrovascular complications [8], patients with type 2 diabetes thus also have to provide comprehensive self-care aiming at control of blood glucose, serum lipid concentrations and blood pressure (BP). Together, these efforts may either induce remission to a non-diabetic state off antidiabetic drugs [9], or test the patients’ motivation to comply with treatment recommendations.

The clinical outcome of type 2 diabetes care is variable, and associated HbA_1c_ values, a surrogate marker of long-term plasma glucose concentration, fluctuate widely and only rarely (~25%) meet therapeutic goals [10]. The associated economic burden is tremendous [11, 12]; increasing with duration of disease, it is estimated to amount to USD 1.31 trillion worldwide [13].

Against that background, it is important to identify both the patients’ potential for metabolic improvement at home, i.e., of the gap between what is and what can be achieved metabolically, and for the possible reduction of associated costs.

To this end, this report describes the potential benefits for patients with type 2 diabetes as to their need for medication, and changes encountered in vital and metabolic variables, risk factors, and costs in response to a 3-week stay at a Diabetes Rehabilitation Clinic (DRC) offering standardized lifestyle, structured diabetes education tailored to their respective treatment mode and individual medical counseling.

## Methods

### Patients and study design

Our observational report aims to generate community-level data required for subsequent clinical trials [14] and to determine the patients’ clinical outcome gap, i.e. their potential for beneficial changes in vital and metabolic variables in response to simple, but standardized changes in lifestyle. To this end, this descriptive pre/post-report analyzes the clinical state of all type 2 diabetes patients (N = 930, aged 57±10 years) admitted consecutively for treatment at our DRC by the Austrian Health Insurance system between June 2013, and June 2016. Analysis took place both at admission and discharge after three weeks.

All referrals were at the request of the respective patients’ physician and were based on the patients’ apparent inability to cope with the needs of proper T2D care at home. A pre/post design was chosen to determine the patients’ clinical outcome gap, i.e. their potential for beneficial changes in vital and metabolic variables, and their associated need for medication during treatment at the DRC.

The study has been reviewed and approved by the Ethics Committee of the Medical University of Vienna (1527/2016) and all participants gave written informed consent prior to inclusion in the study, none declining to participate or dropping out.

### Definitions

Type 2 diabetes is defined according to the American Diabetes Association’s criteria [6], with co-morbidities like hyperlipidemia defined as serum cholesterol >200 mg/dL (5.17 mmol/mol), hypertension as arterial BP >140/90 mmHg or mean arterial BP (MAP, diastolic BP plus BP-amplitude/3) >107 mmHg, and obesity according to body mass index (BMI, [kg/m^2^]). Remission of type 2 diabetes is defined as absence of any need for anti-diabetic medication associated with an HbA_1c_ <6.5% (category 1), while improvements in HbA_1c_ are termed category 2 if values reached 6.5 to 7.0%, and as category 3 when they were reduced to ≥7.0%.

### Treatment

Lifestyle: at the hotel like setting of the DRC, patients were exposed to a standardized lifestyle offering three meals a day low in salt (5 mmol/d) and rich in vegetables and fruits, totaling 1,200–1,600 kcal/d, and to bouts of exercise, such as hiking, swimming, or gymnastics, equivalent to an additional energy expenditure of 400–600 kcal/day. This was done without specifically monitoring patient compliance. An educational intervention was realized by seminars on type 2 diabetes care (WW, HF), individual counseling by physicians (WW, HF, EH), dieticians and diabetes educators, and by peer pressure.

Medication: anti-diabetic treatment was either by i) diet alone, or, if required, by diet plus ii) glucose-lowering drugs other than insulin (GLD [doses/day]: i.e. sulfonylurea, glinides, metformin, DPP-4 inhibitors, SGLT-2 inhibitors, or GLP-1 receptor agonists), and/or iii) insulin [U/(kg.d)], and iv) titrated to target (blood glucose: fasting, 6.1 mmol/l; and 1 h postprandially, <10.0 mmol/l) [6] with preference for metformin and DPP-4 inhibitors.

Additional medication is documented as the number of tablets ingested per day for lipid-lowering drugs (statins), antihypertensives (ACE inhibitors, angiotensin-II receptor blockers [ARBs], diuretics, calcium antagonists, beta-blockers, or alpha-blockers), antidepressants, and for any other medication.

### Examinations

Medical history documents diagnoses, duration of type 2 diabetes, medications, frequency of past hypoglycemic events (symptomatic or BG <50 mg/dl; N/week), co-morbidities, and also smoking habits and compliance with recommendations for influenza and pneumococcal immunization [15] as an indirect measure of the patients’ compliance with health and treatment recommendations at home.

Physical examination evaluates biometric variables (BMI, waist circumference [cm], BP, and MAP [mmHg]), and lipodystrophies in patients treated with insulin. Mood is rated by use of the WHO-5 well-being index, a score of <52% being indicative of depression [16], and diabetic neuropathy by using a neuropathy symptom (NSS) and deficit (NDS) score ranging from 0 (normal) to 10 (severe) [17].

Blood samples were drawn at admission and two days before discharge and analyzed at the MVZ für Laboratoriumsmedizin, Germany, applying ISO 15189 accredited procedures. This also included measurements of HbA_1c_, whose changes in response to intervention are a continuous process and can already be seen as early as after two weeks [18].

### Statistical analyses

Continuous data are given as means±standard deviations or as median (Quartile 1; Quartile 3). Categorical data are listed as counts and percentages.

Paired continuous data are compared by Wilcoxon test or Student’s t-test, as appropriate. Correlations between continuous data are calculated according to Pearson. Categorical data are compared by Pearson’s χ^2^ tests or McNemar’s tests.

Improvements in vital/metabolic outcome after three weeks of hospitalization are expressed as relative reduction of the respective values vs. those at admission, and calculated by binary logistic regression models, whereas goodness-of-fit is evaluated by interpreting areas under the curve (AUC) of receiver operating characteristic (ROC) plots. Analytically relevant differences between HbA_1c_ concentrations at baseline and discharge are defined as changes exceeding the reference change value (RCV): [19]

$$RCV=Z*\sqrt{2*({CV}_{A}^{2}+{CV}_{I}^{2})}$$

Given a Z (Z-score) of 1.96 for significance at 95% probability level, a CV_A_ (analytical coefficient of variance) of 1.05% and a CV_I_ (individual biological variation) of 1.9%, [20] a relative difference >6% in HbA_1c_ concentration between baseline and discharge was considered diagnostically relevant.

Framingham HARD CHD (coronary heart disease) risk scores [21], a measure of the risk of myocardial infarction or a stroke within the next ten years, are reported as relative and absolute changes vs. baseline at discharge.

P-values are recalculated according to Benjamini/Hochberg and considered significant if <0.05. All calculations are done using SPSS 23 (IBM, Armonk, USA), Prism 6 (GraphPad Software, La Jolla, USA) and MedCalc version 15.8 (MedCalc Software, Ostend, Belgium).

## Results

### Participants

Baseline characteristics of patients with type 2 diabetes (age 57±10 years; females 42%; mean duration of disease 8±8 years; age at manifestation 41±26 years) at admission to the DRC (Table 1) show male sex to be associated with higher prevalence of active and former smokers (54%) than female sex (37%; p<0.0001).

**Table 1:** Patient characteristics at baseline.

	Median (IQR) or counts (%)		p-Value	
	Total	Male	Female	
**Sex – no. (%)**	-	539 (58%)	391 (42%)	**0.0001**
**Age, yr**	57±10	56±10	58±10	>0.05
**Medical history**				
**Age at onset of disease (yr)**	41±26	42±24	39±29	>0.05
**Duration of type 2 diabetes (yr)**	8±8	8±7	8±8	>0.05
**Smokers** -active	201 (22%)	124 (23%)	77 (20%)	**<0.0001**
-former	230 (25%)	165 (31%)	65 (17%)	
**Vaccination** -influenza	82 (9%)	49 (9%)	33 (9%)	>0.05
-pneumococci	47 (5%)	23 (4%)	24 (6%)	>0.05
**Treatment**				
-Diet	186 (20%)	98 (18%)	88 (23%)	>0.05
-GLD	690 (74%)	413 (77%)	279 (71%)	>0.05
*- OADs*	690 (74%)	413 (77%)	277 (71%)	>0.05
*- GLP-1 receptor agonists*	29 (3%)	12 (2%)	17 (4)%	>0.05
-insulin, without GLDs	54 (6%)	28 (5%)	24 (6%)	>0.05
-insulin, with GLDs	178 (19%)	105 (20%)	75 (19%)	
**Diabetes-associated complications**				
-Lipodystrophy/infiltrations*	77 (39%)	42 (32%)	35 (35%)	>0.05
-Hypoglycemias/week *- OADs alone*	0 (0; 0)	0 (0; 0)	0 (0; 0)	>0.05
*- insulin*, *without OADs*	0 (0; 0)	0 (0; 0)	0 (0; 1)	>0.05
*- insulin*, *with OADs*	0 (0; 0)	0 (0; 0)	0 (0; 0)	>0.05
-Neuropathy				
*- NSS*	0 (0; 3)	0 (0; 2)	0 (0; 3)	>0.05
*- NDS*	2 (0; 3)	2 (0; 3)	2 (0; 2)	>0.05
-Nephropathy				
*- creatinine (mg/dL)*	0.9±0.3	1.0±0.3	0.8±0.2	**<0.0001**
*- GFR >90 (ml/min/1*.*73m^2^)*	49%	51%	179 (48%)	>0.05
*- GFR 60–89*	41%	41%	40%	
*- GFR 30–59*	9%	7%	12%	
*- GFR 15–29*	1%	1%	1%	
*- GFR <15*	<1%	<1%	<1%	

Characteristics of patients with type 2 diabetes obtained at admission to the DRC grouped according to sex. Data are given as means ± SD, median (IQR), or counts (%). GLDs, glucose-lowering drugs; OADs, oral anti-diabetic drugs

*, insulin requiring patients only.

Differences between sexes are also apparent in the use of oral anti-diabetic drugs, which is somewhat less in females, while their rate of insulin-mediated hypoglycemia seems to be more frequent than that seen in males. No differences were seen between males and females as to modes of diabetes care, rate of diabetes-associated complications, and rate of vaccinations against influenza (9%) and pneumococci (5%).

**Fig 1:**
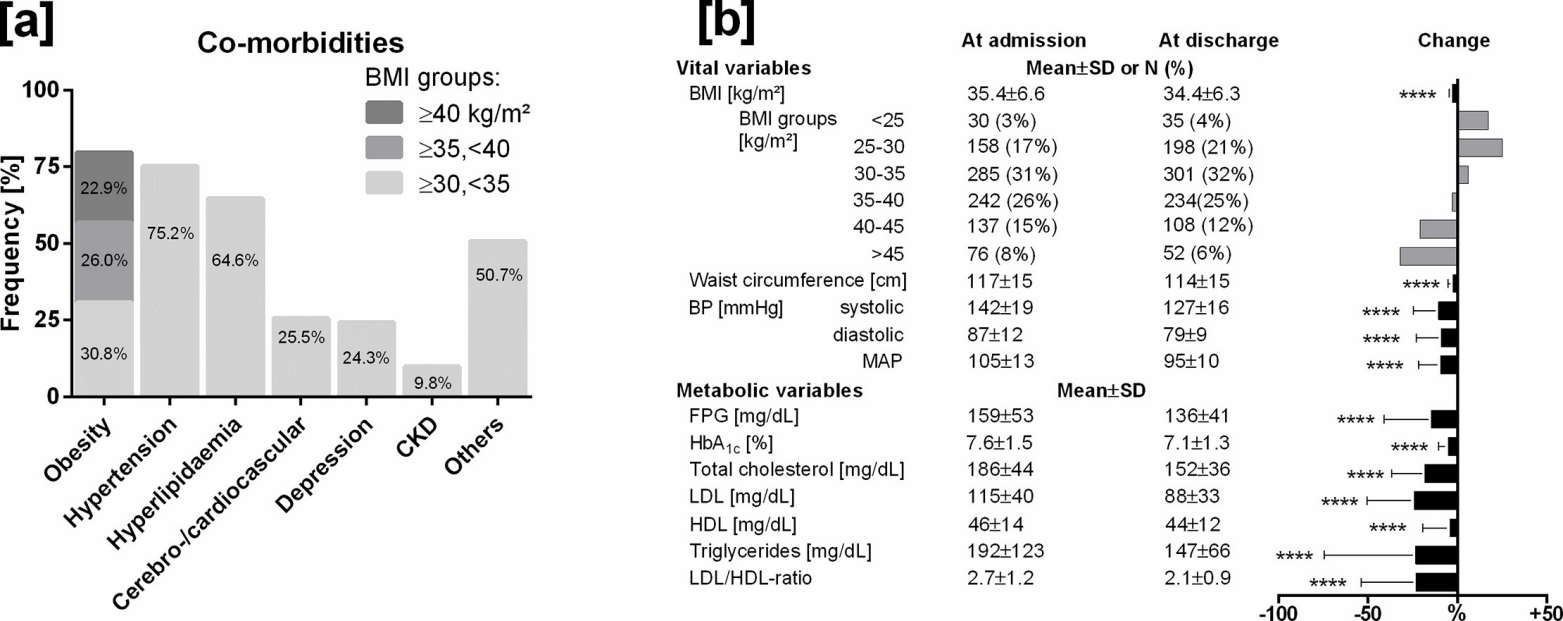
[a] Prevalence of co-morbidities. CKD, chronic kidney disease. [b] Vital and metabolic variables at baseline and their changes at discharge after three weeks. BP, blood pressure; MAP, mean arterial pressure; FPG, fasting plasma glucose. To convert values to mmol/L, multiply by 0.056 (glucose), by 0.026 (total cholesterol, LDL, HDL) and by 0.011 (triglycerides). p-values are derived from paired Student’s t-tests. ****, p<0.0001.

Co-morbidities (Fig 1A) were present in 96% of patients and were dominated by obesity (79%), followed by arterial hypertension (75%), hyperlipidemia (65%), cardio-/cerebrovascular diseases (25%), and depression (24%), while impaired kidney function (GFR<60ml/min/1.73m^2^) was apparent in 10%.

Vital and metabolic variables at baseline (Fig 1B) for the whole group of patients with type 2 diabetes show different values between males and females occurring only for BMI (males=35.0±6.1 vs. females=36.1±7.1, p<0.05), waist circumference (119±15 vs. 115±16, p<0.001), and blood lipids (total cholesterol 182±44 vs. 191±44, p<0.01; LDL 112±39 vs. 119±40, p<0.01; HDL 44±14 vs. 50±14, p<0.0001; LDL/HDL ratio 2,8±1,2 vs. 2.6±1.1, p<0.05; triglycerides 202±120 vs. 174±119, p<0.01).

Analysis of type 2 diabetes care across the country on the basis of HbA_1c_ values obtained at admission and the patients’ respective postal area code unveils no regional differences in the patients’ inability to reach therapeutic targets (F=1.83, p>0.05). Additionally, no differences are apparent at baseline if HbA_1c_ values are compared on the basis of their attending physicians’ subspeciality as either general practitioners or diabetes specialists.

### Effects of standardization of lifestyle

Re-analysis of the clinical state at discharge pertained to all patients with type 2 diabetes included in this observational report, as all of them completed the 3-week course offered.

Vital and metabolic variables: significant reductions in response to three weeks of lifestyle and treatment modification (p<0.0001) were seen both in mean vital (BMI, -3%; waist circumference, -3%; MAP, -10%) and metabolic variables (HbA_1c_, -7%; LDL/HDL ratio, -23%) (Fig 1B).

Of note, smokers, consuming 20 (10; 20) cigarettes per day for 30 (25; 40) years (≙26 [16; 40] packyears), and ex-smokers (≙25 [13, 44] packyears) showed larger waist circumferences (both, +3±18 cm; p<0.05) and higher serum triglyceride concentrations (smokers, +40±100; ex-smokers: +16±102 mg/dl. p<0.01) than non-smokers. As a consequence, smokers (-64±97 mg/dl) and ex-smokers (-50±97 mg/dl) presented at discharge with a more pronounced drop in serum triglycerides than non-smokers (-35±97 mg/dl, interaction: p<0.01). The decision of ex-smokers to stop smoking was commonly due to previous major health hazards and taken prior to and not after admission to the DRC.

The decrease seen in MAP applied to both patients with (72%; -9±13 mmHg; p<0.0001) and without (25%; -12±13 mmHg, p<0.0001) antihypertensive treatment as well as to those with newly prescribed antihypertensive medication (3%; -13±13 mmHg, p<0.0001, interaction: p = n.s.).

The same pattern applied to changes at discharge of LDL/HDL ratio, which fell both in patients with (52%; -0.56±0.80, p<0.0001) and without (16%; -0.45±0.80, p<0.0001) pre-established statin therapy as well as in those with newly established statin therapy (32%; -0.66±0.81, p<0.0001, interaction p = n.s.).

Antidiabetic medication: the drugs used by patients with type 2 diabetes at admission were dominated by glucose-lowering drugs (74%; OADs and GLP-1 receptor agonists) and insulin (25%), 77% of these patients receiving GLDs as add-on medication (Table 1), while only 20% were on a diet alone.

**Fig 2:**
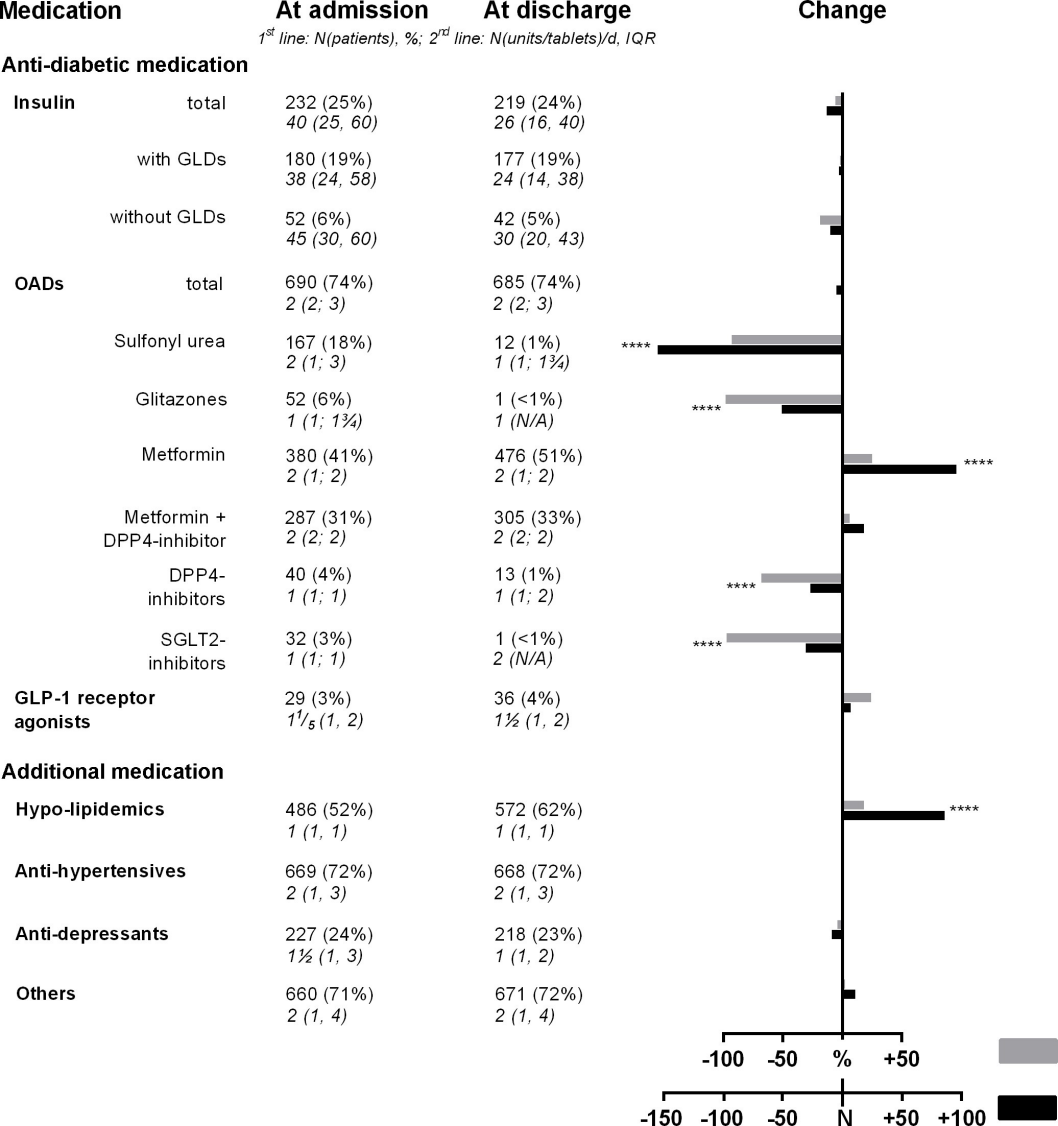
Medication (antidiabetic, hypo-lipidemic, antihypertensive, antidepressant, others) at admission and discharge (grey: relative change from baseline, black: absolute change). p-Values are derived by McNemar tests assessing changes in the number of patients under treatment for each entity. GLDs, glucose-lowering drugs other than insulin; OADs, oral antidiabetic drugs; ****, p<0.0001.

At admission, the majority of type 2 diabetes patients taking OADs were on metformin either alone (380 [41%]) or in combination with DPP-4 inhibitors (287 [31%]), while 40 (4%) used DPP-4 inhibitors only. Furthermore, some patients also received sulfonylureas (167 [18%]), glitazones (52 [6%]), or SGLT-2 inhibitors (32 [3%]), mainly as add-on medication, all of which could be almost completely withdrawn during the patients’ stay at the DRC, and could be replaced in part by metformin either alone (+19%) or in combination with DPP-4 inhibitors (+23%) (Fig 2).

Among patients on insulin, daily insulin dose (U/day) could be reduced during hospitalization by almost 40% from 0.38 units/KG_body weight_ (0.23; 0.55) to 0.23 units/KG_b.w._ (0.13; 0.41; p<0.0001), and omitted in 36 patients (5%), permitting a more pronounced BMI decrease at discharge (-0.9±0.7) than in those without reduction of insulin dose (-0.5±0.7, both p<0.0001. Interaction: p<0.01).

In contrast, insulin treatment had to be initiated anew in 23 (3%) and daily insulin dosage had to be increased in 36 type 2 diabetic patients (5%) by 0.04 units/KG_b.w._ (0.01; 0.07). Improvement of glycemic control incurred in insulin-treated patients was identical to that in those receiving GLDs only and independent from the applied mode of insulin therapy (interaction: p = n.s.).

Additional medication: at admission, additional treatment of patients with type 2 diabetes was with hypo-lipidemic drugs (52%, N = 486), antihypertensives (72%, N = 669), antidepressants (24%, N = 227), and with other medications (71%, N = 660). Among those, hypo-lipidemic drugs had to be terminated in 14 patients due to muscle pain, and was initiated in 100 at the DRC (p<0.0001), while the share of patients receiving antihypertensives, antidepressants, or any other pharmacological treatment (N = 660, 71%) did not markedly change between admission and discharge (Fig 2).

Interestingly, the number of patients with type 2 diabetes receiving antidepressants at admission was four times greater than the Austrian average (males 6.7%, females 10.9%) and depend neither on age (p = n.s.), duration of illness (p = n.s.), nor on the mode of anti-diabetic treatment (p = n.s.).

### Individual benefit and potential for improvement

**Fig 3:**
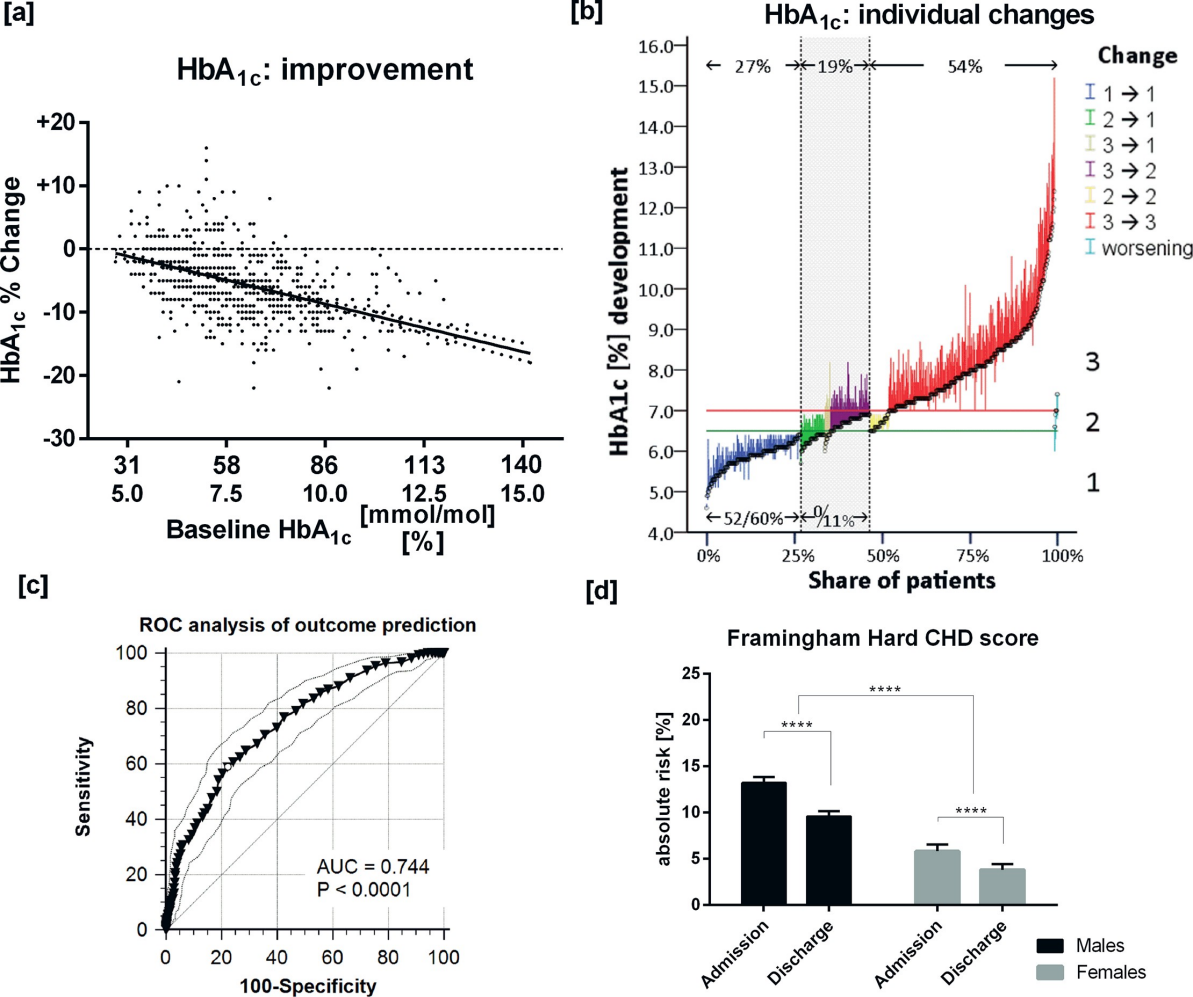
Changes in HbA_1c_ levels (%) from baseline in response to three weeks at the DRC. [a] Dependence of relative improvement on initial HbA_1c_ concentration; [b] individual changes in HbA_1c_: vertical lines denote the respective changes in HbA_1c_ incurred at discharge (vs. at admission) by single patients with type 2 diabetes, and black circles values reached at discharge showing 27% to stay below and 8% to reach an HbA_1c_ <6.5%, while in 11% HbA_1c_ decreased to <7.0%, i.e. to therapeutic target. Patients between dotted vertical lines improved HbA_1c_ by at least one category (1: <6.5; 2: 6.5 - <7.0; 3: >7.0%). *Bottom*: share of the respective subgroup (%) in complete remission (HbA_1c_ <6.5% without any anti-diabetic medication) at admission vs. at discharge. [c] Prediction of HbA_1c_ outcome (decrease > 6% of baseline values) by baseline HbA_1c_ levels; and [d] improvement of the Framingham Hard CHD risk score (means ± 95% confidence interval; subgrouped for sex) in response to three weeks at the DRC. **** p<0.0001.

Patients with type 2 diabetes showing high HbA_1c_ values at admission responded to the standardization of lifestyle with a significantly higher relative decrease in HbA_1c_ than those with lower values (r=-0.485, p<0.0001; Fig 3A), as previously shown by others [22].

Of note, after three weeks at the DRC, 322 patients (35%) had HbA_1c_ values below 6.5%, and the percentage of those in complete remission without any anti-diabetic medication rose from 14% at admission to 18% at discharge. Additionally, 117 patients (13%) with initial HbA_1c_ levels ≥7.0% improved their HbA_1c_ by at least one category to 6.5-<7.0%, or even to <6.5%. The majority of patients with HbA_1c_ >8.0% at admission followed suit and improved their metabolic state considerably, although the allotted time of three weeks was too short for reaching therapeutic targets (Fig 3B).

Regarding the prediction of relevant changes in HbA_1c_ from baseline HbA_1c_ in response to three weeks at the DRC (area under the curve [AUC]=0.744, p<0.0001. Fig 3C), Youden’s index was maximal (J=0.37) at HbA_1c_ >7.7%, showing a sensitivity of 59% and a specificity of 78%. If the cut-off was reduced to HbA_1c_ >6.9%, sensitivity increased to 79%.

In parallel, the Framingham HARD CHD risk score fell between admission and discharge from 9.5±7.2% to 6.7±6.2% (p<0.0001). As male individuals started from a higher level than females (13.2±7.1% vs. 5.8±7.1%), they experienced greater reduction in HARD CHD risk (-3.6±4.7% vs. -2.0±4.7%, p<0.0001; Fig 3D).

Though HbA_1c_ at baseline correlated well with duration of type 2 diabetes (ρ=0.479, p<0.0001), improvements of HbA_1c_ at discharge were slightly more pronounced only in patients with duration of disease >10 years (-0.47±0.44 vs. -0.40±0.44, interaction: p<0.05), but not in those with duration of disease ≤10 years. In parallel, the latter displayed a marginally more marked reduction in BMI (-1.1±0.7 vs. -0.8±0.7, interaction: p<0.001), systolic BP (-16±20 vs. -12±20, interaction: p<0.05), diastolic BP (-9±12 vs. -6±12, interaction: p<0.05), and MAP (-11±13 vs. -8±13, interaction: p<0.05), whereas no differences were seen between the two groups in their changes in waist circumference, total cholesterol, LDL, HDL, LDL/HDL ratio and triglyceride outcomes (interactions: p = n.s.).

Interestingly, analysis by general linear models shows individual benefits accrued by patients with type 2 diabetes at the DRC to be independent of their personal health care provider, i.e. a general practitioner or a specialized diabetes outpatient service (HbA_1c_: p = n.s., LDL/HDL: p = n.s., BMI: p = n.s.) suggesting identical quality of their care at home.

### Costs

Costs per day at the DRC including board, lodging, physical rehabilitation, and expenses for anti-diabetic and supplementary medication are modest (€131) when compared to those incurred in standard (acute) hospitals, ranging from 594 to 2,042 euros/day (see S1 Table).

In addition, costs spent on medications per day fell due to a reduction in insulin and OAD use by ~€ 221 for the total study cohort (see S2 Table).

## Discussion

Analysis of the outcome of type 2 diabetes care after three weeks at our DRC shows considerable reduction in surrogate metabolic markers of diabetes care (HbA_1c_ -0.4±0.4%; LDL/HDL ratio -0.6±0.8; triglycerides -45±98mg/dl), in BMI (-1.0±0.7 kg/m^2^), blood pressure (MAP -10±13 mmHg), and an increase in the share of patients in remission from 14% to 18% in response to standardized, but simple changes in lifestyle. The reduction in the Framingham HARD CHD risk score by 29% is similar to that previously described in patients with plain obesity [23] and occurred in parallel to a simultaneous diminution in the use of insulin by 35% and of OADs other than metformin and DPP-4 inhibitors up to 99%.

In contrast to type 1 diabetes care, which benefits greatly if offered by diabetes specialists at home [24], the outcome of type 2 care at home appears, across the country, to be independent of the patient’s provider, be it a general practitioner or diabetes specialist, as shown by coupling HbA_1c_ values to patients’ respective postal area codes and their respective providers medical qualifications.

The observed outcome of type 2 diabetes care in response to three weeks at a DRC is remarkable when compared with that of trials reporting reductions in HbA_1c_ by 0.43 to 1.79% upon long-term OAD exposure [25], and debunks the contention that type 2 diabetes commonly requires complex forms of treatment. In addition, our findings support the observation that intensive lifestyle interventions reducing daily calorie intake as described in the LOOK AHEAD [26] and DiRECT studies [9], as well as interventions by bariatric surgery [27, 28], promote considerable improvement of metabolic control and reduce the need for anti-diabetic medications [29]. We thus argue against the uncritical use of GLDs in the absence of sufficient patient compliance with basic treatment rules of type 2 diabetes care.

The improvement of integrated type 2 diabetes care seen in patients exposed to three weeks’ modest lifestyle modification suggests that benchmark values of treatment [6] could also be reached at home more frequently by simple means, and thereby help to close the gap between what is and what could be achieved metabolically if people were to re-standardize their eating habits and to increase their physical activity in the course of their lives. To this end, patients and their advisors would, however, have to internalize the notion that treatment of type 2 diabetes relies strongly on control of energy balance, i.e. of calorie intake and energy expenditure (physical exercise), which cannot be offset by add-on anti-diabetic medication. Without such adherence to basic treatment guidelines, successful type 2 diabetes care will continue to face an almost insurmountable challenge [30].

The importance of adherence to the basic rules of diabetes care, including lifestyle adaptation, has been stressed previously both for patients with type 1 diabetes [24] and for those with type 2 diabetes [26], whose glycemia particularly benefits in response to intensive calorie restriction [31] and weight loss [9]. These findings are in line with the improvements of both vital (BMI -3%; MAP -10%) and metabolic variables (HbA_1c_ -7%; LDL/HDL ratio -22%) seen in this study in response to multifactorial treatment of patients with type 2 diabetes independent of their pre-established treatment mode. This is important, as such integrated diabetes care reduces the clinical risk of cardiovascular events and associated mortality [5, 32].

Of note, baseline HbA_1c_ values over 7.7% describe the group of patients with type 2 diabetes benefitting most from a DRC course. The sensitivity of such approach can be further increased if the HbA_1c_ cut-off is lowered to 7.0%, as the then resulting patient group covers almost 80% of all those benefitting from the program.

The use of antidepressants by 24% of our patients, exceeding two- to four-fold the prevalence of depressive disorders among the local population (males 6.7%, females 10.9%), reflects an odds ratio of 1.80 for depression in patients with type 2 diabetes [33] and suggests a considerable burden of disease.

Overall, this pre/post report shows that it is commonplace for patients at home to display inadequate compliance with standards of type 2 diabetes care. Such neglect extends to recommended *i*) avoidance of smoking, *ii*) effective attention to co-morbidities such as hypertension and hyperlipidemia, which ought to be treated properly, and *iii*) influenza and pneumococcal immunization [15].

Any such disregard for treatment recommendations is detrimental in type 2 diabetes patients, particularly if they suffer from co-morbidities supporting the progression of diabetic micro- and macrovascular complications [34]. This is regrettable, as the occurrence of diabetic complications can be minimized by aiming closely for benchmark values of diabetes care [35].

Gender differences with respect to the proportion of smokers, the use of anti-diabetic agents and the somewhat higher rate of hypoglycemia per week might suggest that women are more risk-averse and more anxious to avoid hyperglycemia than men.

Besides its beneficial effects for the patients themselves, type 2 diabetes care at a DRC also comes with an economic advantage, as its costs per day amount only to 15 to 25% of that encountered in standard hospitals (see S1 Table) frequently accepting the same patients with HbA_1c_ above the therapeutic target. In this context, it is of note that patients sustaining HbA_1c_ levels <7.0% (53 mmol/mol) for >3 years also require less financial support (−$5,214.--/3 years) than those with HbA_1c_ ≥7.0% (53 mmol/mol) [36]. Likewise, a recent economic evaluation revealed the average annual direct costs of type 2 diabetes care (including complications) to exceed the annual costs of maintaining diabetes remission within the DiRECT/Counterweight-Plus intervention program [37].

Limitations applying to this report include that, firstly, the setting at the DRC does not allow for untreated control groups and randomization, as patients are admitted primarily for treatment of their clinical condition. Second, compliance with treatment recommendations remained the patient’s choice. Non-compliance with treatment recommendations at the DRC was, however, seemingly rare, as failure to reduce overweight or HbA_1c_ was only seen in 2.3% and 10.4% of our type 2 diabetes patients respectively. Thirdly, potential distortion of data by regression to the mean is unlikely, as both patients with initially extremely high HbA_1c_ levels and almost all of those with HbA_1c_ levels below the therapeutic threshold reduced their respective HbA_1c_ values after three weeks at the DRC.

Although the outcome of this pre/post-observational study cannot be extrapolated long-term, since it only describes what can be achieved during a short period of time, it nevertheless demonstrates that the clinical state of patients with type 2 diabetes benefits greatly by proper adaptation of lifestyle.

Furthermore, our report suggests that any attempt to close the clinical and metabolic outcome gap between what is and what can be achieved in type 2 diabetes care requires all parties involved to *i*) regard food intake, exercise, and medication as inseparable, intertwined entities, and *ii*) accept that such integrated care cannot be replaced simply by some add-on glucose-lowering drugs. To this end, it appears that *‘imprinting’* patients accordingly at a DRC could be of considerable help in meeting treatment goals.

## Supporting information

S1 Table Costs of hospitalization [€/d and patient; range] depending on hospital type in Austria. Data: Parliamentary records 891/AB XXV. GP, https://www.parlament.gv.at/PAKT/VHG/XXV/AB/AB_00891/imfname_349174.pdf, and https://www.akhwien.at/default.aspx?pid=789. GLDs, glucose-lowering drugs; €, Euro; ICU, intensive care unit.
(DOCX)

S2 Table Savings in daily treatment costs in response to three weeks at the DRC for the total cohort. (DOCX)
