# Supplementary material for: Type 2 diabetes care: Improvement by standardization at a diabetes rehabilitation clinic. An observational report
Source: PLoS One. 2019 Dec 12;14(12):e0226132. doi: 10.1371/journal.pone.0226132 (PMC6907777; doi:10.1371/journal.pone.0226132)
Supplement: S1 Table — Data: Parliamentary records 891/AB XXV. GP, https://www.parlament.gv.at/PAKT/VHG/XXV/AB/AB_00891/imfname_349174.pdf, and https://www.akhwien.at/default.aspx?pid=789. GLDs, glucose-lowering drugs; €, Euro; ICU, intensive care unit. (DOCX) [file pone.0226132.s001.docx]

|  | **Costs/day** [range] |
| --- | --- |
| **Hospitalization** |  |
| **- University hosp.** | € 1,145.-- |
| **- Acute** [non university] **hospital** | € 682.--  [594 – 816] |
| **- Acute hosp.,** ICU | € 1,813.--  [1,351 – 2,042] |
| **Rehabilitation Clinic** | € 131.-- |

**S1 Table. Costs of hospitalization [€/d and patient; range] depending on hospital type in Austria.** Data: Parliamentary records 891/AB XXV. GP, https://www.parlament.gv.at/PAKT/VHG/XXV/AB/AB_00891/imfname_349174.pdf, and <https://www.akhwien.at/default.aspx?pid=789>. LDs, glucose-lowering drugs; €, Euro; ICU, intensive care unit.
