# Supplementary material for: Type 2 diabetes care: Improvement by standardization at a diabetes rehabilitation clinic. An observational report
Source: PLoS One. 2019 Dec 12;14(12):e0226132. doi: 10.1371/journal.pone.0226132 (PMC6907777; doi:10.1371/journal.pone.0226132)
Supplement: S2 Table — (DOCX) [file pone.0226132.s002.docx]

|  | **Admission** | **Discharge** | **Δ** |
| --- | --- | --- | --- |
|  | (costs/total cohort, €/day) | | |
| **Insulin** | 394.53 | 241.36 | 153.17 |
| **OAD**  **Sulfonyl urea** | 85.28 | 3.90 |  |
| **Glitazones** | 59.15 | 0.91 |  |
| **Metformin** | 43.59 | 57.09 |  |
| **MetComb** | 638.88 | 717.53 |  |
| **DPP4-Inhibitors** | 114.66 | 39.78 |  |
| **SGLT2-Inhibitors** | 91.26 | 4.68 |  |
| **GLP1-receptor agonists** | 147.32 | 287.98 |  |
| **Total** | **1574.67** | **1353.23** | **221.44** |

**S2 Table. Savings in daily treatment costs in response to three weeks at the DRC for the total cohort.**
